# Complete genome sequences of three *Bradyrhizobium* strains isolated from root nodules of peanut (*Arachis hypogaea*) cultivar Tainan Select No. 9 collected in Taiwan

**DOI:** 10.1128/mra.01413-25

**Published:** 2026-02-27

**Authors:** Shen-Chian Pei, Ya-Yuan Hsu, Sook-Kuan Lee, Chih-Yun Chen, Chi-Te Liu, Chih-Horng Kuo

**Affiliations:** 1 Institute of Plant and Microbial Biology, Academia Sinica 71559, Taipei, Taiwan; 2 Department of Agronomy, National Taiwan University 124715, Taipei, Taiwan; 3 Institute of Biotechnology, National Taiwan University 607377, Taipei, Taiwan; 4 Department of Horticulture, National Chung Hsing University 593979, Taichung, Taiwan; 5 Biotechnology Center, National Chung Hsing University 593851, Taichung, Taiwan; University of Strathclyde, Glasgow, United Kingdom

**Keywords:** *Bradyrhizobium*, genome, peanut, Taiwan, nodule

## Abstract

We report complete genome sequences of three *Bradyrhizobium* strains isolated from root nodules of peanut cultivar Tainan Select No. 9 collected in Taiwan. Hybrid assemblies using Nanopore and Illumina reads produced circular chromosome and plasmid contigs, providing high-quality genomic resources for future studies.

## ANNOUNCEMENT

Peanut-associated *Bradyrhizobium* strains are involved in nodulation and nitrogen fixation, thereby supporting the growth of their plant host (1). To facilitate bioresource development, we report the complete genome sequences of three *Bradyrhizobium* strains isolated from peanut root nodules collected in Taiwan.

The workflow followed previous studies (2, 3), kits were used according to the manufacturers’ instructions, and bioinformatics tools were used with default settings. These strains were isolated previously (4). Briefly, peanut cultivar Tainan Select No. 9 (TNS9) was collected in June 2015 from National Taiwan University (25.0153°N 121.5406°E). Fresh root nodules from individual plants were surface sterilized using 70% ethanol and 2% sodium hypochlorite for 5 min, washed three times with sterile water, and crushed. The exudate was streaked onto laboratory-prepared yeast extract mannitol (YEM) agar plates (5) and maintained at 30°C in darkness for 5 days. Single colonies were restreaked on fresh YEM agar plates at least three times and stored as glycerol stocks at −80°C.

The strains were revived by streaking on YEM agar plates under the same incubation conditions. Single colonies were cultured in 5 mL of YEM broth at 30°C in the dark with shaking at 220 RPM for 5–6 days until OD_600_ reached 1. Cells were collected by centrifugation at 16,000 × *g* for 1 min at 4°C, then processed using Nanobind CBB kit (102-301-900; PacBio) for DNA extraction. The same DNA samples were used for both sequencing platforms. For Oxford Nanopore Technologies (ONT) sequencing, samples were processed using the ONT Ligation Sequencing Kit (SQK-NBD114-96) without shearing or size selection and sequenced on a MinION (FLO-MIN114). Base calling was performed using Dorado v7.6.8 with super-high-accuracy mode and a minimum *q*-score of 10. For Illumina sequencing, samples were processed using NEBNext Ultra II DNA Library Prep Kit (New England Biolabs) and sequenced on a NovaSeq X Plus using the NovaSeq X Series 25B Reagent Kit (300 cycles) to obtain paired-end 150 bp reads.

For hybrid *de novo* assembly, ONT reads longer than 1,000 bp were combined with Illumina reads that were trimmed using Trimmomatic v0.39 (6) and assembled using Trycycler v0.5.5 (7). For validation, ONT and Illumina reads were mapped to the contigs using Minimap2 v2.15-r905 (8) and BWA v0.7.17-r1188 (9), respectively. The mapping results were checked using SAMtools v1.9 (10) to assess sequencing depth. For each strain, Trycycler directly assembled two large circular contigs corresponding to the chromosome and a plasmid. Genome completeness was further supported by CheckM2 v1.1.0 (11), which estimated 100% completeness and <3% contamination. For species identification, assemblies were compared to reference genomes in the NCBI RefSeq database (12) using FastANI v1.33 (13). To identify chromosomal *dnaA* and plasmid-borne *repA*, protein sequences from reference genomes were used as TBLASTN v2.11.0 (14) queries against each assembly. The circular contigs were rotated to have these as the first genes. Assemblies were submitted to GenBank and annotated using the NCBI Prokaryotic Genome Annotation Pipeline v6.10 (15). Key information is summarized in Table 1.

**TABLE 1:** Genome assembly statistics for the three strains^*a*^

Strain	T-1	T-2	T-6
Species assignment	*Bradyrhizobium* sp.	*Bradyrhizobium guangdongense*	*Bradyrhizobium guangdongense*
Reference assembly and strain	GCF_018130615.1 (SZCCT0344)	GCF_004114975.1 (CCBAU 51649^T^)	GCF_004114975.1 (CCBAU 51649^T^)
Alignment fraction (%)	78.6	94.3	95.1
Average nucleotide identity (%)	99.0	98.3	98.2
Nanopore reads accession; read count; *N*_50_; combined length; sequencing depth	SRR36097091; 52,101; 33,463 bp; 1,055,045,192 bp; 113.6×	SRR36097093; 69,275; 28,531 bp; 1,230,550,786 bp; 149.8×	SRR36097395; 51,749; 34,633 bp; 1,065,645,335 bp; 133.1×
Illumina reads accession; read count; combined length; sequencing depth	SRR36097092; 41,345,002; 6,243,095,302 bp; 514.6×	SRR36097094; 35,823,830; 5,409,398,330 bp; 600.9×	SRR36097396; 38,045,748; 5,744,907,948 bp; 576.4×
Assembly accession	GCA_053592855.1	GCA_053669695.1	GCA_053669755.1
Chromosome size (bp)	8,237,534	7,173,377	6,972,587
Chromosome GC content (%)	63.5	63.8	63.9
Plasmid size (bp)	981,918	979,288	979,288
Plasmid GC content (%)	59.3	59.3	59.3
Total number of annotated genes	8,802	7,818	7,660
Number of intact protein-coding sequences	8,509	7,514	7,377
Number of pseudogenes	235	250	229
Number of complete rRNA genes (5S, 16S, 23S)	1, 1, 1	1, 1, 1	1, 1, 1
Assembly completeness and contamination estimates (%)	100.0, 1.25	100.0, 0.46	100.0, 2.62

^*a*^ Species assignment followed the >95% ANI criterion relative to the designated reference genome.

## Data Availability

The sequencing reads and genome assemblies have been deposited in NCBI GenBank and are publicly available; accession numbers are listed in Table 1.
